# A molecularly imprinted polymer as solid phase extraction sorbent for ketoprofen determination in water and artificial serum prior to HPLC

**DOI:** 10.55730/1300-0527.3485

**Published:** 2022-07-19

**Authors:** Elif GÜREL ÖZYURT, Ömer ÖZYURT, Yekta Arya ÖLÇER, Ahmet Emin EROĞLU, Ezel BOYACI, Talal SHAHWAN

**Affiliations:** 1 Department of Chemistry, İzmir Institute of Technology, İzmir, Turkey; 2 Department of Materials Science and Engineering, İzmir Institute of Technology, İzmir, Turkey; 3 Department of Chemistry, Middle East Technical University, Ankara, Turkey; 4 Department of Chemistry, Birzeit University, Ramallah, West Bank, Palestine

**Keywords:** Ketoprofen, molecular imprinted polymers, solid phase extraction, high performance liquid chromatography, NSAIDs

## Abstract

Ketoprofen (KET) is an active pharmaceutical compound that has pain relieving and antipyretic effects. Its determination in body fluids and environmental waters is important due to widespread use of the compound. In this study, a selective and reliable method has been developed for the determination of ketoprofen in water and artificial serum using molecularly imprinted polymers (MIPs) as a solid phase extraction sorbent prior to HPLC-DAD detection. The MIP was synthesized by copolymerization of methacrylic acid (MAA) and trimethylpropane trimethacrylate (TRIM) in the presence of ketoprofen as the template. For the sake of comparison, nonimprinted polymer (NIP) was also synthesized under the same experimental conditions without the addition of ketoprofen under the same experimental conditions. Critical extraction parameters such as sample pH, shaking time and sorbent amount were optimized and adjusted to 8.0, 24 h, and 10.0 mg, respectively, for a sample volume of 10.0 mL. MIP showed higher selectivity than NIP towards ketoprofen in an artificial matrix containing another pain relieving drug, ibuprofen, and a cardiovascular drug, metoprolol. The proposed method was successfully applied for the detection of ketoprofen in spiked drinking water, tap water, and artificial serum samples, and showed satisfactory results with respective recoveries of 96.8 % (± 0.8), 93.7% (± 0.6), 62.2% (± 0.6), and 69.9% (± 0.6).

## 1. Introduction

Nonsteroidal antiinflammatory drugs (NSAIDs) are commonly used in human and veterinary medicine as reducers of pain and inflammation caused by diseases. These drugs are also considered endocrine disrupting compounds (EDCs) because of their adverse effects on mammalian body. Endocrine disrupting compounds are exogenous chemicals that interfere with and disrupt functions of hormones [1]. They increase the incidence of breast cancer and anomalies in the human reproductive system. Widespread consumption of these medicals makes them important environmental pollutants. It is reported that NSAIDs are not efficiently removed in wastewater treatment plants and can be found in surface waters at trace levels [2,3]. Several studies showed that NSAIDs may have adverse effects on aquatic organisms and humankind [4,5]. Ketoprofen (KET) is among the propionic acid derivatives under NSAIDs. It blocks the activity of cyclo-oxygenase (COX) enzymes that catalyze the formation of prostaglandin [6,7]. Ketoprofen is widely used for the treatment of musculoskeletal inflammation and other inflammatory conditions. In a 24-h period, approximately 80% of an administered dose of ketoprofen is excreted in urine, primarily as the glucuronide metabolite. The side effects of ketoprofen are not only encountered during the exposure to the drug, but can also be experienced through the use of contaminated environmental waters. It is therefore crucial to detect ketoprofen in different matrices such as environmental water and body fluids. Several analytical methods have been developed for the determination of ketoprofen in complex aquatic matrices [8]. Generally, it is determined by chromatographic techniques including gas chromatography [9], high performance liquid chromatography [10], and capillary electrochromatography [1,11]. Detection of ketoprofen in the aquatic environment is difficult due to its trace amounts, and complex matrices of waters which necessitate the application of sample pretreatment step(s) prior to chromatographic analysis [12].

Solid phase extraction (SPE) is commonly used for the enrichment and/or separation of target analyte(s) from sample solution. Various sorbents can be used in SPE processes including molecularly imprinted polymers (MIPs). MIPs are obtained for specific or selective recognition of target analytes by copolymerization of crosslinking agents and functional monomers. Imprinting sites are created by covalent or noncovalent interaction between a functional monomer and a template molecule during prepolymerization step. After polymerization, the template molecule is removed from the polymer network. The resulting polymer usually possess cavities that are specific to an analyte or selective to a group of analytes. The specificity or selectivity is determined during the selection of a template molecule. Although the interaction between the analyte and the functional site in MIP is chemical rather than physical, selectivity is usually obtained by choosing the template molecule that has the largest molecular size in its group. If the template molecule is the smallest one in the group, the other molecules are not expected to fit into the cavities created and then the specificity is achieved. In addition to their selectivity and specificity, MIPs are preferred due to their low cost, ease of preparation in a short time, having high stability and activity during a wide range of conditions and robustness [7, 13, 14]. The SPE method in which MIPs are used as sorbents is called molecular imprinted solid-phase extraction (MISPE) [15,16].

In this work, MIP was synthesized for the selective recognition of ketoprofen in several sample types prior to HPLC-DAD analysis. Experimental parameters including solution pH, sorbent amount, shaking time, and desorption solvents were investigated. The proposed MIP-SPE method was applied to spiked waters and artificial serum samples.

## 2. Experimental

### 2.1. Materials and solutions

All chemicals and reagents were of analytical reagent grade, unless stated otherwise. Ketoprofen (KET, ≥98%), ibuprofen (IBU, ≥98%), metoprolol (MET, ≥98%) were obtained from Sigma Aldrich. Standard solutions were prepared in methanol (1000.0 mg L^−1^) and stored at −20 °C. HPLC grade acetonitrile (ACN) and methanol (MeOH) were purchased from Sigma Aldrich. For chromatographic analysis ultrapure water was used (18.2 MΩ, Millipore). In the synthesis of MIPs, methacrylic acid (MAA, ≥99%, Sigma Aldrich), trimethylolpropane trimethacrylate (TRIM, Aldrich), and 2,2’ azobis(2,4-dimethyl) valeronitrile (AIVN, Alfa Aesar) were used.

### 2.2. Instrumentation and apparatus

Chromatographic analyses were carried out using Agilent 1200 Series HPLC (Agilent Technologies) with diode array detector [9]. Separations were performed at room temperature with a Supelco C18 HPLC column (Lichrosphere RP 18-5, 25 cm, 4.6 mm). The mobile phase composition was MeOH:H_2_O (80:20). In the preparation of the mobile phase, pH of the water was adjusted to 3.0 with acetic acid and then added to MeOH in all cases. The flow rate was 0.9 mL min^−1^, the absorbance measurements were taken at 220 nm and injection volume was 20.0 μL. The zeta potential of MIP/NIP were determined at 25 °C by using a zetasizer (Micromeritics Particulate Systems). Surface area, total pore volume and average pore diameter for both MIP and NIP were evaluated using Micromeritics Gemini V BET analyzer.

### 2.3. Synthetic approach for preparation of ketoprofen-imprinted polymers

**Figure 1:**
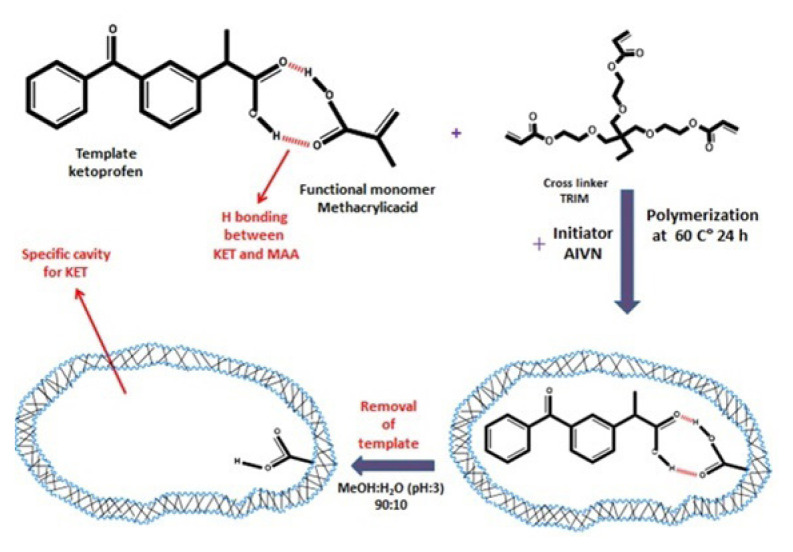
Schematic representation of the synthesis of MIP via copolymerization of MAA and TRIM.

The MIP particles were synthesized via precipitation polymerization. The synthesis procedure was similar to the procedure outlined in a previous study with some modifications [7]. Various template/monomer/crosslinker mole ratios were tried as 1/4/10, 1/2/20, and 1/8/20 and it was found that 1/8/20 was determined to be the optimum as given in subsection 3.2. The details of the synthesis procedure are as follows. Firstly, 1.0 mmol ketoprofen and 8.0 mmol MAA were dissolved in 200.0 mL ACN. For prepolymerization, the mixture was stirred for 1.0 h at room temperature during which the H-bonding between MAA and ketoprofen was formed. Then, 20.0 mmol TRIM was added into the reaction mixture. The initiator (AIVN) was used in a concentration corresponding to 2.0% of total moles of MAA and TRIM [7]. The mixture was purged with nitrogen gas for 10 min to remove oxygen. Polymerization was performed in an oil bath with constant stirring at 60.0 °C for 24 h. After the polymerization process, the template molecule ketoprofen was removed from the solid using MeOH:H_2_O (80:20) (pH of the water adjusted 3.0 with acetic acid) mixture. Following the template removal, MIP was dried in an oven at 60.0 °C. The nonimprinted polymer (NIP) was synthesized under the same conditions without the addition of ketoprofen into the polymerization mixture. A schematic representation of the synthesis procedure is given in Figure 1.

### 2.4. Characterization of the synthesized polymers

The surface morphology of MIP was investigated through scanning electron microscopy (SEM). The Brunauer–Emmet–Teller (BET) model was used to evaluate specific surface area, total pore volume, and average pore diameter for both MIP and NIP.

### 2.5. Sorption and selectivity studies

The sorption performances of the synthesized MIP and NIP were demonstrated by binding characteristic assay. For this purpose, solutions at different ketoprofen concentrations (1.0 to 250.0 mg L^−1^, 10.0 mL) were prepared from stock solution by appropriate dilutions. Into each of these solutions, 10.0 mg MIP/NIP were added and the mixtures were shaken at 560 rpm for 24 h. At the end of the shaking period, the mixtures were filtered and ketoprofen concentrations in the solutions were determined by HPLC-DAD.

To show if the recognition of MIPs towards ketoprofen is selective, the sorption of MIP/NIP was performed in presence of metoprolol and ibuprofen (each at 1.0 mg L^−1^) by keeping the other parameters constant (10.0 mL sample volume, 10.0 mg MIP/NIP, 24 h shaking at 560 rpm).

In addition to the selectivity test described above, the distribution coefficients *K*_d_ (mL g^−1^) of the analytes were calculated using Eq. 1, where *C_0_* is the initial concentration of analyte (mmol L^−1^) before sorption, and *C_e_* (mmol L^−1^) stands for the final concentration of the analyte remaining in the liquid phase after sorption. W and V represent the mass (g) of the polymer and the volume (mL) of the solution, respectively. The selectivity coefficient for the sorption of ketoprofen in the presence of ibuprofen and metoprolol was calculated using Eq. 2. The relative selectivity coefficient (K′) was determined using Eq. 3 to compare the selectivity of MIP and NIP [17].


(1) $$K_{d}=\frac{\left(C_{0}-C_{e}\right)}{WC_{e}}V$$

(2) $$K=\frac{K_{d}(\text{ketoprofen})}{K_{d}(\text{competitir})}$$

(3) $$K^{\prime }=\frac{K(\mathrm{MIP})}{K(\mathrm{NIP})}$$

### 2.6. Sorption isotherm models

Sorption isotherms are employed to model the equilibrium distribution of the analyte between the solid and liquid phases at various concentrations and constant temperature, and thus describe the mobility of the analyte in the medium [17]. Various isotherm models have been applied for this purpose. Langmuir, Freundlich, and Dubinin–Radushkevich isotherms are the most widely used models. The Langmuir model assumes that sorption occurs at homogeneous sorption sites with a monolayer coverage, and that the energy of sorption is independent on the extent of sorption. The nonlinear form of Langmuir isotherm is given in Eq. 4.


(4) $$Q_{e}=Q_{\max}\frac{bC_{e}}{1+bC_{e}}$$

Here, *Q*_max_ (mmol g^−1^) and b (L mmol^−1^) are Langmuir constants; *Q*_max_ is the amount of sorbed ketoprofen corresponding to monolayer coverage, b is the affinity of ketoprofen for the sorbent, C_e_ (mmol L^−1^) is the amount of ketoprofen in liquid phase at equilibrium, and *Q*_e_ is the amount of ketoprofen sorbed on the surface of the sorbent (mmol g^−1^) at equilibrium. The values of isotherm constants are evaluated from the linearized form of the equation, which is given in Eq. 5.


(5) $$\frac{1}{Q_{e}}=\frac{1}{Q_{\max}}+\frac{1}{Q_{\max}bC_{e}}$$

The intercept and the slope of the plot of *1/Q_e_* vs. *1/C_e_* were used in the determination of *Q*_max_ and b [18].

Freundlich isotherm is applied for heterogeneous surfaces, over a wide range of analyte concentrations. In this model, sorption is not limited to a monolayer coverage, and thus sorption can be nonlinear, i.e. the driving force of sorption is affected by surface coverage. The nonlinear form of this isotherm is described by Eq. 6 [19].


(6) $$Q_{e}=K_{F}{C_{e}}^{1/n}$$

Here, K*_F_* and n are constants which are characteristic for a given sorbent-sorbate system, under a fixed set of experimental conditions. K*_F_* reflects the sorption capacity, while *1/n* is related to sorption linearity. These constants can be evaluated from the linearized form of Eq. 6 which is given in Eq. 7.


(7) $$\log Q_{e}=\log K_{F}+\frac{1}{n}\log C_{e}$$

The intercept and the slope of the plot of *logQ*_e_ versus *logC*_e_ in Eq. 7 give *logK*_F_ and *1/n*, respectively.

Dubinin–Radushkevich (D-R) isotherm model applies usually for intermediate concentration ranges and assumes that the analyte binds preferentially to energetically favorable sites of a sorbent, with the possibility of multilayer sorption [17]. D-R isotherm is described by Eqs. 8–10.


(8) $$Q_{e}=q_{s}\exp\;(-B{ɛ}^{2})$$

(9) $$\text{where }\ln Q_{e}=\ln q_{s}-B{ɛ}^{2}$$

(10) $$\text{here }ɛ=\mathrm{RT}\;\ln(1+\frac{1}{c_{e}})$$

In D-R model, *q_s_* (mg g^−1^) corresponds to the maximum sorption monolayer capacity, B (mol^2^ kJ^−2^), gives information about the mean free energy of sorption per molecule of sorbate which is required to transfer it to the surface of the solid from infinity in the solution. The mean free energy of sorption *E* can be calculated from *B* parameter using Eq. 11.


(11) $$E={\left(2B\right)}^{-1/2}$$

The constants *q_s_* and *B* are calculated from the intercept and slope of the linear plot *lnQ*_e_ of versus *ɛ*^2^.

### 2.7. Optimization parameters

The effect of various experimental parameters of SPE, such as sample pH, sorbent amount, desorption solvent, sorption time and reusability of the sorbent, was studied by keeping all the parameters constant except the investigated one. All the sorption procedures were performed using an orbital shaker at 560 rpm. Effluents and eluents were analyzed by HPLC-DAD.

Firstly, the effect of solution pH on extraction was investigated. For this purpose, the pH of the solutions was adjusted to 4.0, 6.0, 8.0, and 10.0 at the ketoprofen concentration of 1.0 mg L^−1^ (10.0 mL). After that, 10.0 mg MIP/NIP were added into sample solutions. In a separate experiment, the effect of solution pH on the extraction of ketoprofen was examined in the presence of metoprolol and ibuprofen. In this study, a 10.0 mL mixture of ketoprofen, metoprolol and ibuprofen (1.0 mg L^−1^ each) were used. The effect of sorbent amount on extraction was investigated by changing it from 5.0 to 100.0 mg for 10.0 mL of 1.0 mg L^−1^ ketoprofen solutions. Following that, the effect of sample volume was investigated for 5.0, 10.0, 25.0, 50.0, and 100.0 mL of sample solutions with 1.0 mg L^−1^ ketoprofen and 10.0 mg sorbents. For the determination of optimum sorption time, the sorption procedure was carried out for up to 24 h (1.0 mg L^−1^ of 10.0 mL ketoprofen solution, 10.0 mg of sorbent). The effect of eluent type was also investigated for desorption of the analyte retained by MIP. For this purpose, 10.0 mL of MeOH, ACN and MeOH:H_2_O (80:20) (pH of the water adjusted 3.0 with acetic acid) mixture were used as eluents, separately. Reusability of the sorbent was checked with repetitive sorption/desorption of 10.0 mL of 1.0 mg L^−1^ ketoprofen solution using 10.0 mg MIPs. After each sorption/desorption cycle, the sorbent was dried in an oven for 1 h at 50 °C. This procedure was successively repeated ten times for the same sorbent.

### 2.8. Calibration and method validation

A stock solution of 1000.0 mg L^−1^ ketoprofen was prepared in methanol. Working standards were prepared by dilution of stock solution. A calibration graph was obtained by measuring standard solutions at different concentrations. The instrumental limit of detection (LOD) and the limit of quantification (LOQ) were calculated by analyzing the least concentrated standard 10 times with HPLC-DAD. The validity of the method was tested via spike recovery studies using ultrapure, drinking and tap water samples spiked with 1.0 mg L^−1^ ketoprofen. The spike recovery tests were also carried out with artificial serum and phosphate buffer saline solutions.

The serum samples were prepared in accordance with the recipe given in Ref. [20], and contained 4.5 mM KCl, 5 mM CaCl2, 1.6 mM MgCl2, 4.7 mM d(+)-glucose, 2.5 mM urea, 0.1 % bovine serum albumin (BSA), and 145 mM NaCl which was also spiked with ketoprofen whenever necessary. In addition, another solution resembling artificial serum was also prepared by adding 6 % bovine serum albumin into phosphate buffered saline (PBS) (pH 7.6).

## 3. Results and discussion

### 3.1. Chromatographic method

Optimum mobile phase composition, flow rate, and column temperature were determined as MeOH:H_2_O (80:20) (pH of the water adjusted to 3.0 with acetic acid), 0.9 mL min^−1^ and 30 °C, respectively. The instrumental calibration plot was linear up to 250.0 mg L^−1^ with a line equation y = 192.8x–1.5803 (R2 = 0.9999). The LOD and LOQ were calculated as 0.023 mg L^−1^ and 0.079 mg L^−1^, respectively.

### 3.2. Preparation and characterization of MIP and NIP

In this study, several template:monomer:crosslinker mole ratios of 1:4:10, 1:2:20, and 1:8:20 were used for polymerization of MIPs and NIPs. No polymerization occurred when 1:4:10 ratio was used. Polymerization was realized with 1:2:20 ratio, but the sorption capacities of MIP and NIP were found to be insufficient. Mole ratio of 1:8:20 was decided to be optimum not only for demonstrating the best sorption performance among all but also for giving the highest sorption difference between MIP and NIP. Figure 1 shows the possible reaction mechanism of MIP via copolymerization of MAA and TRIM.

Generally, two different approaches, namely covalent or noncovalent, may be employed based on the interaction between the template and the functional monomers during the prepolymerization step [21]. In this study, a noncovalent strategy (through hydrogen bonding) was followed. This approach is more practical due to easy preparation of template monomer noncovalent interactions. In addition, the removal of the template from the polymer network is easier, compared with the covalent approach [22].

### 3.3. Characterization

**Figure 2:**
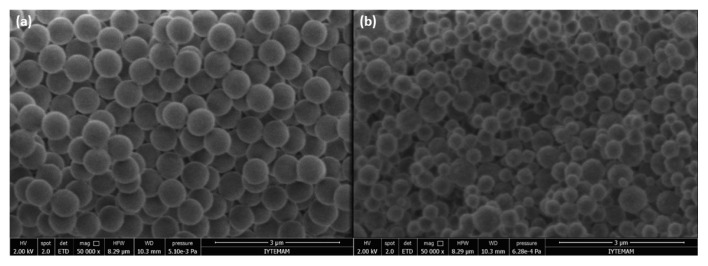
SEM images of the (a) MIP and (b) NIP beads magnified 50,000 times.

The shape and morphology of the synthesized polymer can be estimated by considering the total monomer/solvent ratio (w/v). If this ratio is smaller than 5%, the precipitation polymerization will be the main route and the polymer will have spherical shape [23]. If the ratio is greater than 5%, the main route will be bulk polymerization and the shape of the polymer is expected to be monolithic. In our experiment this ratio was calculated as 2.08 % which resulted in MIP and NIP particles having the spherical shape as expected (Figure 2) [7,24]. As can be seen from the SEM images, MIP particles were more homogeneous than the NIP, which is expected to offer an advantage especially for potential flow-through type of separations (e.g., columns, SPE cartridges). The average particle diameters were determined with ImageJ software for MIP as 511 (±7) nm and for NIP as 369 (±109) nm.

Brunauer–Emmet–Teller (BET) surface area analysis method was used to investigate the surface and porosity of the synthesized polymers. The BET results are given in Table 1, and as indicated, surface area, total volume and pore size of MIP are similar to those of NIP. Even though MIP and NIP have similar results from BET analysis, the MIP could adsorb much more ketoprofen than NIP due to the presence of selective imprinted cavities and regular placement of functional monomers in the surface pores of MIP during the polymerization process.

### 3.4. Sorption and selectivity studies

**Figure 3:**
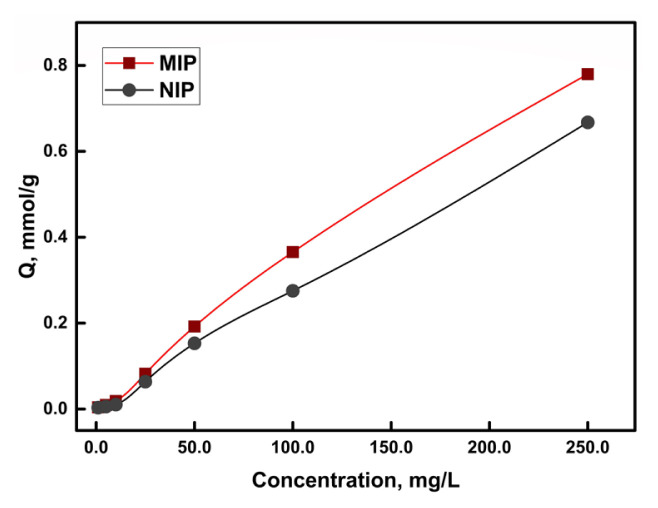
Sorption of ketoprofen with MIP and NIP (10.0 mL sample volume, 10.0 mg sorbent amount, n = 3).

The sorption capacities of MIP and NIP for ketoprofen were determined via rebinding (sorption) experiments. As seen in Figure 3, there is only little differences between MIP and NIP at low concentrations. This result possibly indicates the presence of nonspecific interactions between analyte molecules and surface sites in both MIP and NIP. The available surface sites in NIP (which does not have the specific template cavities) can be sufficient for low analyte concentrations. On the other hand, the capacity difference between MIP and NIP becomes more evident at higher analyte concentrations, as lesser amounts of the analyte are extracted by the NIP compared to MIP sorbent. This can be attributed to the selective/specific sorption sites (template cavities) created in MIP structure. This observation is not only important in terms of the achieved capacity increase, but also in terms of the selectivity gained towards the analyte(s).

**Figure 4:**
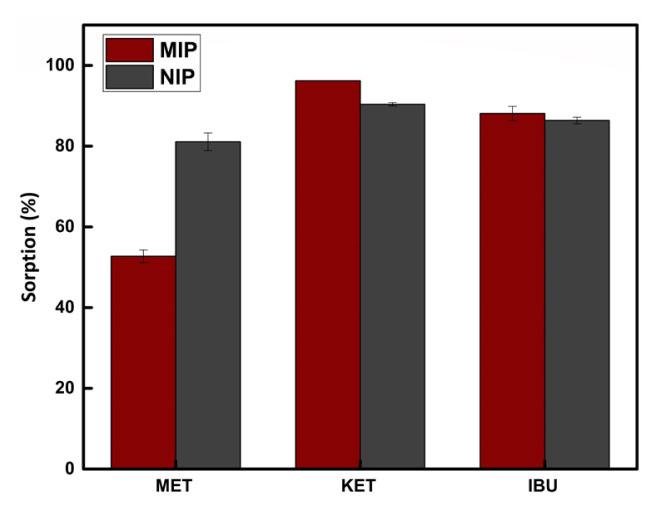
Crossselectivity of MIP and NIP (sample volume 10.0 mL, sorbent amount 10.0 mg, pH 8.0, n = 3).

The selectivity of MIP to ketoprofen was investigated in the presence of the competing ibuprofen and metoprolol molecules (both at 1.0 mg L^−1^ concentration), which can be found in similar environments together with ketoprofen. As seen in Figure 4, under the applied experimental conditions, MIP showed high selectivity also to ibuprofen, a structurally related compound. Ibuprofen is smaller than ketoprofen in size and could possibly be sorbed by the selective cavities easily due to the presence of functional sites that are expected to form hydrogen bonds with the carboxyl group of ibuprofen. For ketoprofen and ibuprofen, MIP has shown slightly higher sorption capacity than NIP due to selective cavities/binding sites. This orientation is not expected to occur for metoprolol (no carboxyl group in its structure) and interestingly, the sorption performance of NIP was better than MIP for metoprolol. It can also be speculated that the higher sorption of NIP than MIP for metoprolol in the presence of ketoprofen and ibuprofen can be attributed to the partial occupation of selective/nonselective binding sites in MIP by ketoprofen and ibuprofen, and thus leaving a smaller number of available sites for metoprolol. All three compounds showed similar sorption behavior with NIP, possibly due to nonspecific interactions.

As described in subsection 2.5, the selectivity coefficients (K) for the sorption of ketoprofen by MIP/NIP was determined in the presence of competing species. Initially, the distribution coefficients (*K*_d_) of ketoprofen, ibuprofen and metoprolol for sorption by MIP and NIP were calculated. The distribution coefficients were used for the determination of the selectivity coefficients for ketoprofen/metoprolol couple (21.53 and 2.33 for MIP and NIP, respectively) and ketoprofen/ibuprofen couple (2.99 and 1.5 for MIP and NIP, respectively). The tabulated values indicate an outstanding relative selectivity of MIP (compared to NIP) to ketoprofen in the presence of metoprolol; namely, 9.24 times more selectivity. The relatively smaller selectivity of MIP (compared to NIP) for ketoprofen in the presence of ibuprofen (only 1.99) can be attributed to the structural similarities of these two compounds, as mentioned previously.

### 3.5. Sorption isotherms

**Figure 5:**
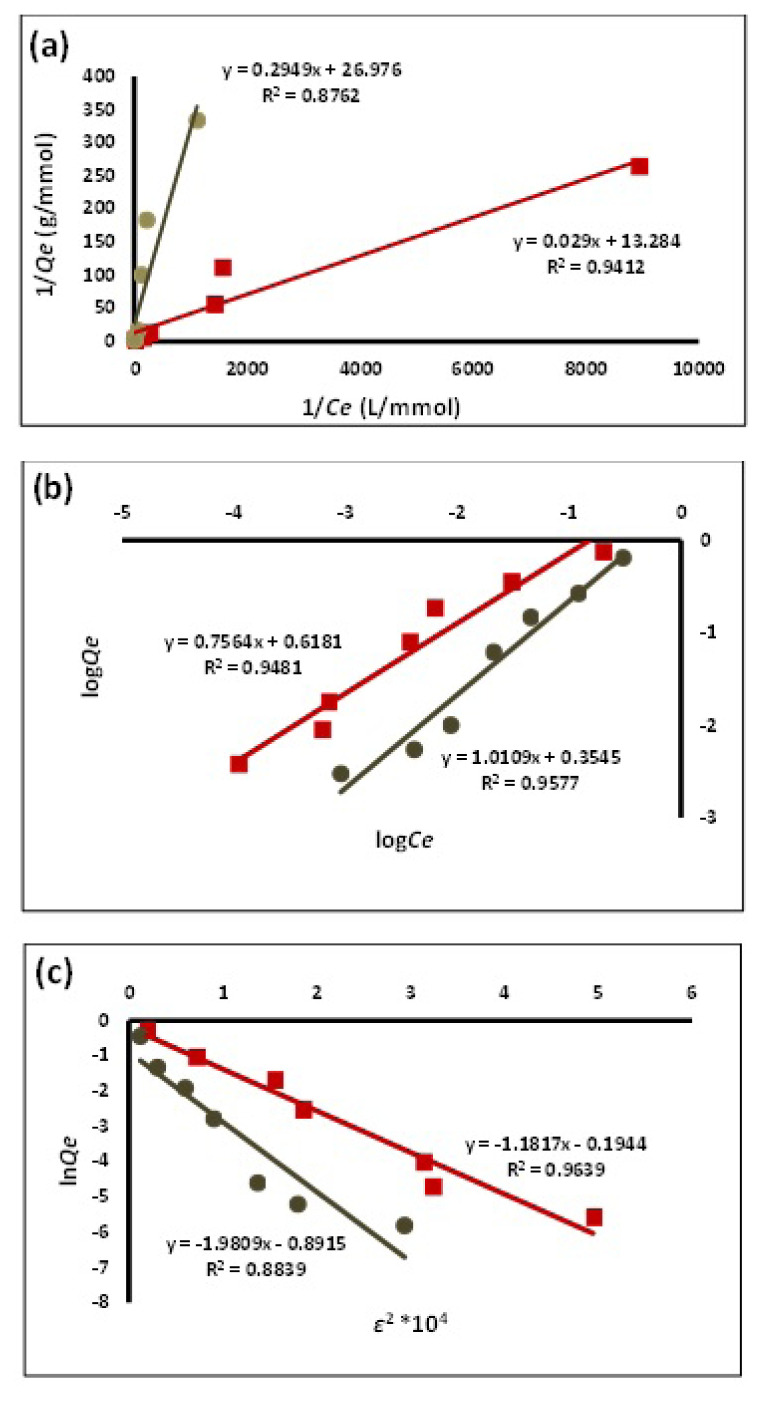
Linear fits of (a) Langmuir, (b) Freundlich, (c) Dubinin–Radushkevich isotherm models for ketoprofen sorption by (■) MIP and (●) NIP.

The linear fits of the isotherm models (Figure 5) were used for the calculation of the isotherm constants, and the results are given in Table 3. In all cases, the isotherm parameters show that the sorption capacity of MIP towards ketoprofen is higher than that of NIP. Based on the coefficient of determination (R^2^), D-R and Freundlich models appear to correlate better with the sorption data of the analyte by MIP, while Freundlich isotherm is clearly the best isotherm for describing the sorption of ketoprofen by NIP. The poorer correlation of Langmuir isotherm may be attributed to the wide concentration range used in this study, which included high initial concentrations that can go beyond a monolayer coverage. On the overall, if one of the three isotherms is to be chosen, as the one with the best correlation with all the sorption data, it would be Freundlich isotherm. This might be suggesting that each of MIP and NIP possesses more than one type of sorption sites, which are different in terms of chemical nature and/or energetic accessibility. It is evident from the values of Freundlich constant, *K*_F_, that the capacity of sorption of MIP is higher than that of NIP [24]. On the other hand, based on the values of *1/n*, the sorption of ketoprofen on NIP appears to be closer to linearity than its sorption on MIP. This indicates that the loss of the driving force for ketoprofen surface coverage is more pronounced in MIP than in NIP, possibly due to the higher extent of sorption of ketoprofen on the former. As it is evident from the isotherm constants of the three models, MIP appears to possess a distinctly higher sorption capacity than NIP.

### 3.6. Optimization of MIP-SPE conditions

**Figure 6:**
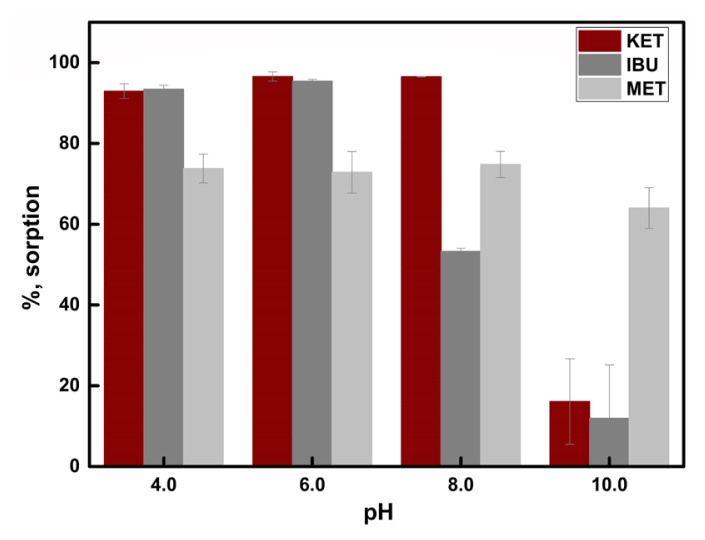
Effect of solution pH on sorption (sample volume 10.0 mL, sorbent amount 10.0 mg, n = 3).

Adjustment of the solution pH can be a crucial step for SPE process since both the performance of the sorbent and the form of the analytes can be affected by pH changes. Also, a change in solution pH may affect the hydrogen bonding between the analyte(s) and the polymer matrix. Therefore, solution pH was changed from 4.0 to 10.0 to investigate the effect of pH on the sorption efficiency of MIP and NIP (Figure 6). MIP showed maximum sorption at pH 8.0 (99.0%, n = 3). Ketoprofen has a pKa value of 5.94; therefore, the molecule must be negatively charged at pH 8.0 (pH 6.5 and above). The percentage sorption of MIP increased with increasing pH at least up to 8.0.[Table t2-turkjchem-46-6-1853]

At pH 6.0 and 8.0, the sorption efficiency of MIP was remarkably higher than NIP. Herein, the surface of NIPs could be deprotonated easily with –OH, but the specific cavities of the MIPs could not be affected much. At pH 10.0, the sorption efficiencies of both MIP and NIP decreased sharply. To find out the reason of this decrease dynamic light scattering (DLS) analysis was made. For pH 4, 6, and 8, the zeta potentials of synthesized MIP and NIP change between −20 and −35 mV (Table 4). This means that the surfaces of MIP and NIP are moderately stable and negatively charged. However, at pH 10, the zeta potentials are even more negative (between −41 and −50 mV) compared to the other pH’s, indicating that the surface is more negative and highly stable against aggregation [25]. Therefore, the limited extent of ketoprofen sorption at pH 10 can be attributed to the repulsive interaction between the negative charged ketoprofen entities and the negatively charged surfaces of MIP and NIP. A similar observation was also reported in another study [26].

**Figure 7:**
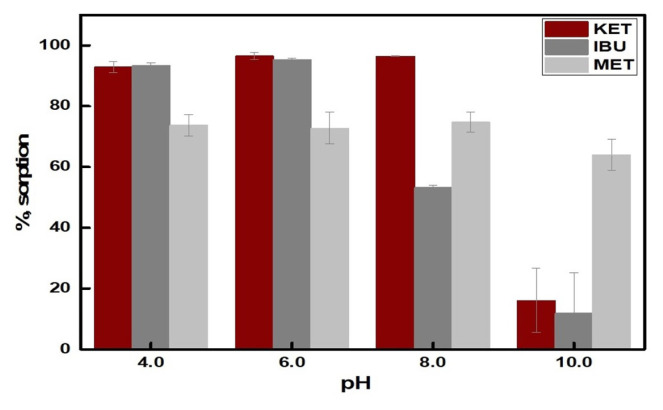
Effect of solution pH on the sorption of ketoprofen in the presence of ibuprofen and metoprolol (sample volume 10.0 mL, sorbent amount 10.0 mg, n = 3).

Furthermore, the effect of solution pH on the specific binding characteristic of MIPs was examined in the presence of ibuprofen and metoprolol. The results are given in Figure 7. At pH 4.0 and 6.0, the sorption affinity of MIP towards both of ketoprofen and ibuprofen was very high, with the two being indistinguishable from each other. This result is consistent with the structural similarities between these two molecules. The sorption percentage for metoprolol was smaller than those of ketoprofen and ibuprofen. At pH 8.0, MIP exhibited higher selectivity to ketoprofen. The difference between the sorption of ketoprofen and ibuprofen can be attributed to their water solubilities. With increasing pH, carboxylic acid groups are converted to carboxylate ions, resulting in an increase in the solubilities of these two compounds in water. Based on the *log*P values of ketoprofen and ibuprofen (3.1 and 3.7, respectively), it might be concluded that the water solubility of ibuprofen exceeds that of ketoprofen, and as such, the hydration forces make the chemical potential of ibuprofen in water smaller than that of ketoprofen, thus leading to a comparatively smaller extent of sorption. At pH 10, there is a sharp decrease in the sorption percentage of MIP towards ketoprofen and ibuprofen as explained in subsection 3.4. However, the sorbent showed relatively higher (around 60%) sorption to metoprolol, possibly due to nonspecific ionic interactions between the negatively charged sorbent surface and the neutral (to partially negative) charge of metoprolol (pKa value of metoprolol is 9.67).

**Figure 8:**
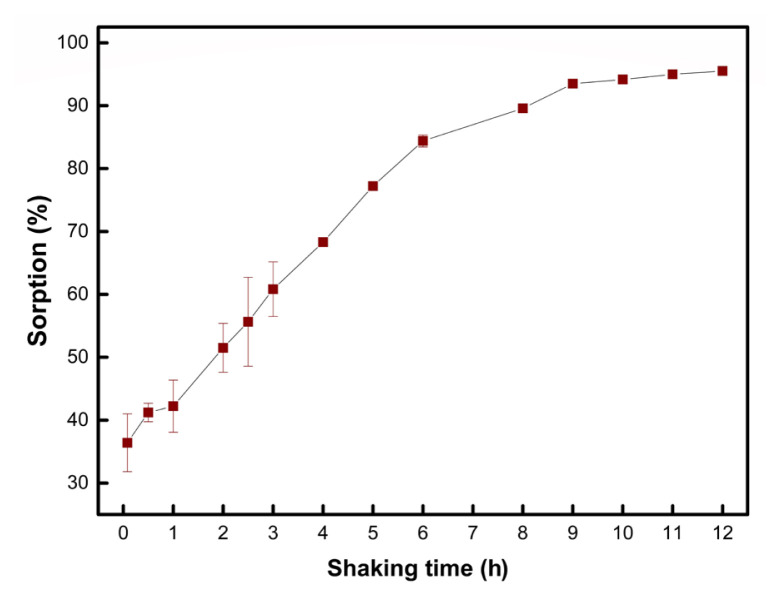
Effect on shaking time on the sorption of ketoprofen (sample volume 10.0 mL, sorbent amount 10.0 mg, n = 3).

The effect of shaking time on the sorption of ketoprofen was also investigated in the study. As shown in Figure 8, the sorption proceeds relatively slowly, as 6 and 12 h are necessary to reach 85% and 95% sorption, respectively. This might be indicative that the sorption process is controlled by diffusion into the sorption sites of the solid matrix. Although the use of more efficient agitation methods can be considered for shorter extraction times, the kinetic aspect of the process is still open to further investigation. In the study, the shaking time was kept at 24 h in all experiments to guarantee quantitative sorption. Use of a proper internal standard is suggested if shaking times shorter than 6 h are to be applied.

The type and composition of the eluent is another important parameter to be considered. The specific interaction between the template (ketoprofen) and the polymer matrix is interrupted with a proper eluent and the previously sorbed analyte is desorbed. In this study, three different eluents, MeOH, ACN and MeOH:H_2_O (80:20) (pH of the water adjusted 3.0 with acetic acid) were used. Among the eluents tested, MeOH:H_2_O (80:20) (pH of the water 3.0) gave the best recovery due to the momentary interruption of the hydrogen bonding between the analyte and the sorbent with acetic acid (Table 5). Therefore, MeOH:H_2_O (80:20) (pH of the water 3.0) was used as the eluent in the remaining experiments. This solvent composition was also employed as the mobile phase in the HPLC-DAD determinations which makes the harmonization of the mobile phase and the eluent unnecessary before the measurements.

**Figure 9:**
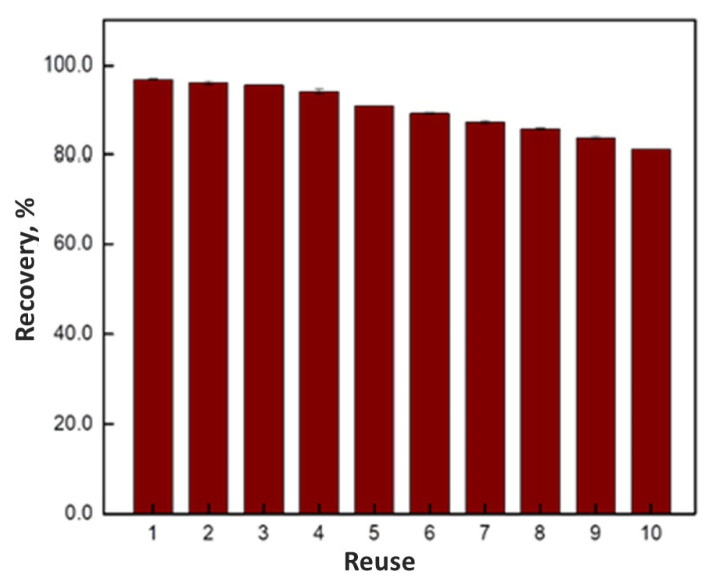
Reusability of MIP-SPE sorbent (sample volume 10.0 mL, sorbent amount 10.0 mg, n = 3).

The reusability of the MIP-SPE sorbent was also investigated and the results are given in Figure 9. As seen, the sorption efficiency remained greater than 85% after eight sorption/desorption cycle. Therefore, the sorbent could be used repeatedly under the same experimental conditions provided that the internal standardization is applied, or alternatively, the sorbent can be used only once due to its easy preparation at an affordable cost.

### 3.7. Method validation

After the optimization of sorption and desorption parameters, the proposed MIP-SPE procedure was applied to several types of water and artificial serum samples spiked with ketoprofen as a part of the method validation.

At this point, it should be mentioned that acidic drugs such as ketoprofen highly bind to albumin, and almost all of the ketoprofen added to the sample will be bound to the protein leaving only a small portion of analyte in the free form (ca 2%). Considering that only the free form of the analyte will be sorbed by MIP, the absolute recoveries will be greatly reduced in this matrix. In order to obtain high recoveries, it is critical to implement a sample pretreatment method which will convert the protein-bound form of the analyte to its free form prior to the MIP-SPE step. Various procedures have been reported that can be used on biological matrix with binding components. Such step will prevent the interferences related to sample matrix by selective precipitation of the macromolecules and increase the free concentration of the analytes. Usually, organic solvents are preferred in the protein precipitation process [27]. In this study, methanol-water (50:50) (v/v) mixture was used to precipitate the albumin from the artificial serum samples, leaving the analytes free in the sample.

Percent recoveries obtained after the application of the MIP-SPE method in different sample matrices are given in Table 6. These results have clearly demonstrated that MIP recoveries were not affected by the matrix-to-matrix variations in different water types and can be used as a reliable method for the determination of ketoprofen. In the case of serum samples, lower recoveries were observed compared to water samples. The main reason for lower recoveries could be related to the matrix modification step performed during the protein precipitation. Considering that an organic solvent (methanol-water) was added to the serum samples for protein precipitation, and that the solvent was not removed from the mixture, this modification must have changed the affinity of the analyte towards MIP. Based on this fact, use of internal standards or matrix-matched calibration is, therefore, recommended if the method is to be used for serum samples.

## 4. Conclusion

In this work, the application of a ketoprofen-imprinted polymer has been proposed for the selective extraction of ketoprofen in different types of water (pure, drinking, and tap) and in artificial serum samples. The MIP was synthesized successfully with methacrylic acid as the functional monomer and ketoprofen as the template.

MIP showed better quantitative sorption than NIP, and was found to be relatively selective to ketoprofen. The selectivity of MIP was also tested in the presence of several other drugs. The results indicate an outstanding relative selectivity of MIP (compared to NIP) to ketoprofen in the presence of metoprolol, and a relatively smaller selectivity of MIP (compared to NIP) for ketoprofen in the presence of ibuprofen, possibly due to the structural similarities of these two compounds. The sorption process of ketoprofen can be described adequately with Freundlich isotherm, and the sorption proceeds relatively slowly, possibly due to the pore diffusion.

After the optimization of experimental parameters, the validity of the proposed method was checked via spike recovery experiments in water (ultrapure, drinking and tap waters) and artificial serum and reproducible recoveries were obtained.

Figure S1 Chromatogram of 1.0 mg L^−1^ mixture solutions of metoprolol (1), ketoprofen (2) and ibuprofen (3). (Mobile phase: MeOH:H2O (80:20) (pH of the water adjusted to 3.0 with acetic acid), flow rate: 0.9 mL min^−1^ and temperature: 30 °C, 220 nm).

## Figures and Tables

**Table 1:** BET analysis results.

Brunauer–Emmet–Teller analysis of polymers			
	Surface area (m^2^ g^−1^)	Total pore volume (cm^3^ g^−1^)	Average pore diameter (nm)
**MIP**	368.9	0.227	2.46
**NIP**	380.8	0.232	2.44

**Table 2 t2-turkjchem-46-6-1853:** Distribution coefficients, selectivity coefficients and relative selectivity coefficients for the MIP and NIP.

Polymer	K_d_/(mL g^−1^)						
	Ketoprofen	Metoprolol	Ibuprofen	K_M_	K_M_’	K_I_	K_I_’
**MIP**	25,088	1165	8386	21.53	9.24	2.99	1.99
**NIP**	9054	3082	6037	2.33		1.50	

**Table 3:** Summary of models’ coefficients and constants.

Adsorption model	Parameter	MIP	NIP
**Langmuir**	Slope	0.029	0.2949
	Intercept	13.284	26.976
	R^2^	0.9412	0.8762
	Q_max_ (mmol g^−1^)	0.0753	0.0371
	b (L mmol^−1^)	458.1	91.5
**Freundlich**	Slope	0.7564	1.0109
	Intercept	0.6181	0.3545
	R^2^	0.9481	0.9577
	K_F_	4.150	2.262
	1/n	0.7564	1.0109
**Dubinin–Radushkevich**	Slope	−1.1817	−1.9809
	Intercept	−0.1944	−0.8915
	R^2^	0.9639	0.8839
	B (mol^2^ kJ^2^)	1.1817 × 10^−4^	1.9809 × 10^−4^
	q_s_ (mmol g^−1^ )	0.823	0.410
	E (kJ mol^−1^)	65.0	50.2

**Table 4:** Zeta potentials of MIP/NIP at different pHs.

Zeta potential, mV				
	pH 4.0	pH 6.0	pH 8.0	pH 10.0
**MIP**	−22.29	−26.20	−27.71	−49.61
**NIP**	−20.48	−22.23	−34.22	−42.04

**Table 5:** Effect of various solvents on the elution of ketoprofen (1.0 mg L^−1^) from MIP.

Eluent	Recovery %
MeOH	47.7 (±1.1)
ACN	76.2 (±2.1)
MeOH:H_2_O (80:20) (pH of the water 3.0)	97.3 (±0.8)

**Table 6:** Recovery results obtained with the proposed method (n = 3).

Sample	Absolute recovery % KET (1.0 mg L^−1^)
Ultrapure water	97.4 (±0.2)
Bottled water	96.8 (±0.8)
Tap water	93.7 (±0.6)
Artificial serum	62.2 (±0.6)
Phosphate buffer saline + BSA	69.9 (±0.6)
