# Supplementary figures and images for: A molecularly imprinted polymer as solid phase extraction sorbent for ketoprofen determination in water and artificial serum prior to HPLC
Source: Turk J Chem. 2022 Jul 19;46(6):1853–65. doi: 10.55730/1300-0527.3485 (PMC10446941; doi:10.55730/1300-0527.3485)

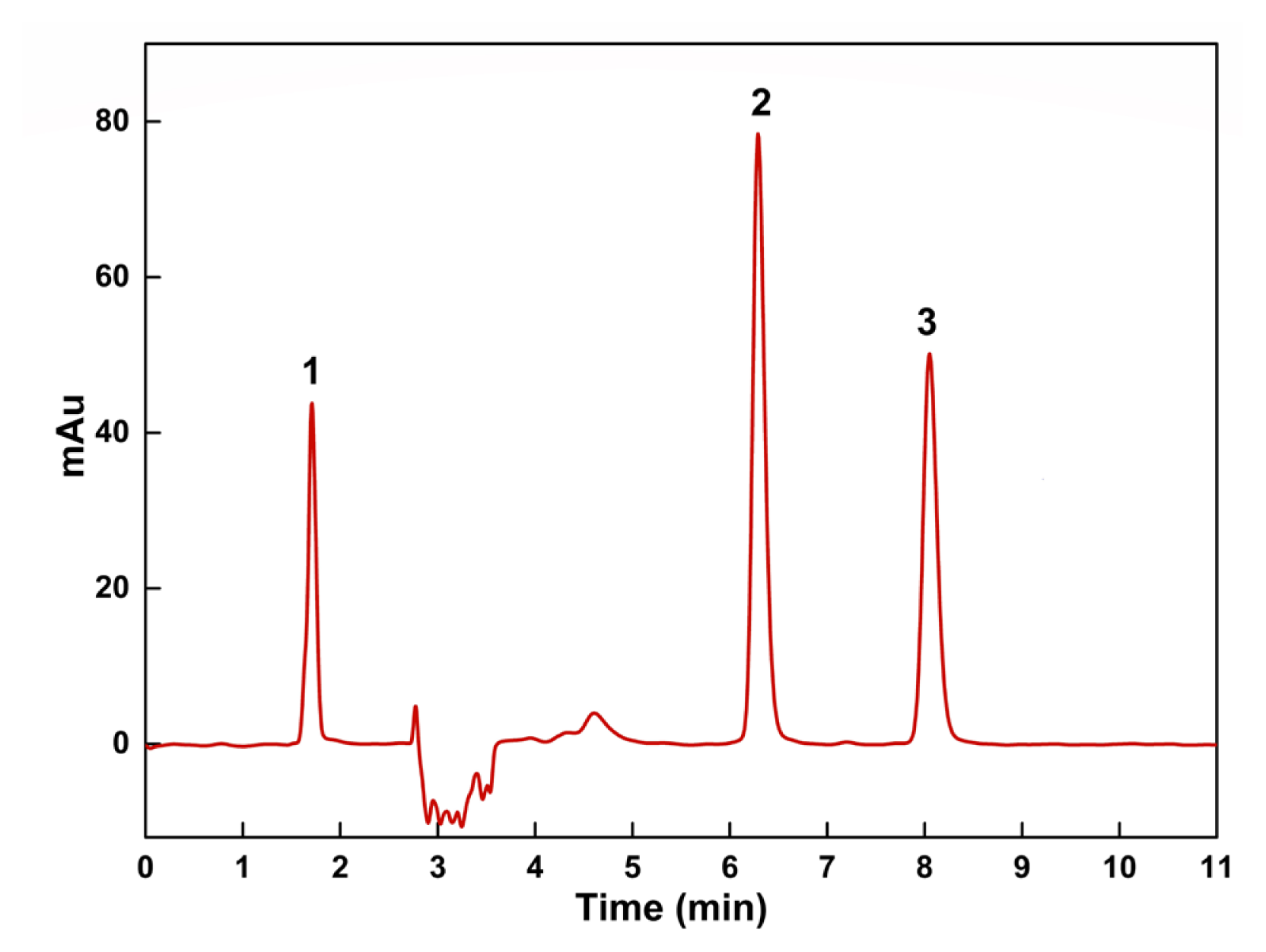

Supplement: Figure S1 — Chromatogram of 1.0 mg L−1 mixture solutions of metoprolol (1), ketoprofen (2) and ibuprofen (3). (Mobile phase: MeOH:H2O (80:20) (pH of the water adjusted to 3.0 with acetic acid), flow rate: 0.9 mL min−1 and temperature: 30 °C, 220 nm). [file turkjchem-46-6-1853s1.tif]
